# Pathologic Complete Response Rates After Neoadjuvant Pertuzumab and Trastuzumab with Chemotherapy in Early Stage HER2-Positive Breast Cancer - Increasing Rates of Breast Conserving Surgery: A Real-World Experience

**DOI:** 10.3389/pore.2021.1609785

**Published:** 2021-05-04

**Authors:** Katalin Boér, Zsuzsanna Kahán, László Landherr, Tibor Csőszi, Károly Máhr, Ágnes Ruzsa, Zsolt Horváth, Barna Budai, Gábor Rubovszky

**Affiliations:** ^1^ Department of Medical Oncology, Szent Margit Hospital, Budapest, Hungary; ^2^ Department of Oncotherapy, Faculty of Medicine, University of Szeged, Szeged, Hungary; ^3^ Department of Oncoradiology, Uzsoki Teaching Hospital, Budapest, Hungary; ^4^ Department of Oncology, Jász-Nagykun-Szolnok Hetényi County Hospital, Szolnok, Hungary; ^5^ Department of Medical Oncology, Zala County Hospital, Zalaegerszeg, Hungary; ^6^ Department of Oncology, Somogy County Kaposi Mór Hospital, Kaposvár, Hungary; ^7^ Oncoradiological Center, Bács-Kiskun County Hospital, Kecskemét, Hungary; ^8^ Department of Molecular Genetics, National Institute of Oncology, Budapest, Hungary; ^9^ Department of Oncology, Faculty of Medicine, Semmelweis University, Budapest, Hungary; ^10^ Department of Thoracic and Abdominal Tumors and Clinical Pharmacology, National Institute of Oncology, Budapest, Hungary

**Keywords:** early breast cancer, HER2+, neoadjuvant systemic therapy, pathological complete response, breast-conserving surgery, neutrophil-to-lymphocyte ratio

## Abstract

**Purpose:** The neoadjuvant use of pertuzumab and trastuzumab with chemotherapy improves the pathologic complete response (pCR) in early HER2+ breast cancer. The aim of this study was to determine the pCR rate obtained with dual HER2 blockade in routine clinical practice. The secondary and tertiary objective was to investigate the impact of neoadjuvant systemic therapy (NST) on performing breast-conserving surgery and survival data.

**Methods:** This was a multicentre, retrospective, observational study in patients with stage II and III HER2+ early breast cancer who received pertuzumab and trastuzumab-based NST. Data were collected from patients’ medical records.

**Results:** Eighty-two patients were included in the study treated in 8 cancer centers in Hungary between March 2015 and January 2020. The study included women with a median age of 50.3 years. The majority of the patients (95%) received a sequence of anthracycline-based chemotherapy followed by docetaxel. pCR was achieved in 54% of the cases. As a result of NST a significant increase of conservative breast surgeries (33% vs. 3.6% planned, *p* = 0.0001) was observed. Ki67 expression and neutrophil-to-lymphocyte ratio (NLR) significantly predicted pCR. None of the variables were independent predictors of DFS.

**Conclusion:** The pCR rate achieved in our study demonstrates the reproducibility of trial data in a real-world population. The rate of breast-conserving surgery was significantly increased.

## Introduction

Neoadjuvant systemic therapy (NST) has many advantages since it serves as a tool for *in vitro* assessment of response, it allows less aggressive surgery, more breast-conserving therapy, and early treatment of micro-metastatic disease [1–4]. Nowadays, anti-HER2 therapy paired with chemotherapy is an essential component of NST in the management of HER2+ early breast cancer (EBC) [5]. In the early 2000s, trastuzumab became an integral part of NST for patients with HER2+ EBC. With the addition of trastuzumab to chemotherapy as NST, a significant improvement in the pCR rate was observed [6–8]. The humanized monoclonal antibody, pertuzumab, binds HER2 at a different site from that of trastuzumab and prevents HER2 dimerization with other HER family members. The primary trial supporting the approval of pertuzumab for use in combination with trastuzumab and docetaxel as NST for patients with HER2+ EBC was the phase II NEOSPHERE trial. In this trial, the results achieved with trastuzumab were improved with the addition of pertuzumab, reaching pCR rates of 39% [9–11]. In neoadjuvant treatments, pertuzumab plus trastuzumab combined with chemotherapy is now recommended as part of the treatment regimen for stage II and III HER2+ EBC [5]. However, treatment patterns and clinical experience of obtaining pCR with dual blockade via pertuzumab and trastuzumab in patients with HER2+ stage II-III EBC have not been assessed extensively in the real-world setting.

This retrospective, observational study of women treated with pertuzumab, trastuzumab and concomitant chemotherapy was aimed to evaluate the achievable pCR rate in real-world clinical practice. We herein present the pCR rate defined as ypT0/is ypN0 obtained with dual HER2 blockade. We also intended to evaluate the rate of conversion from mastectomy to breast-conserving surgery after NST. A third objective was to investigate survival data and prognostic factors.

## Methods

Patients were retrospectively identified from hospital records. Data were extracted from patient medical records to complete an electronic case report form and stored on an anonymous database for analysis. Patients from 8 Hungarian hospitals with HER2+ EBC, who received neoadjuvant dual HER2 blockade, trastuzumab and pertuzumab in addition to chemotherapy from March 2015 to January 2020 were included for analysis. This retrospective observational study included women with HER2+, operable (T2–3, N0–1, M0), or locally advanced (T2–3, N2–3, M0 or T4a–c, any N, M0), or inflammatory (T4d, any N, M0) breast cancer, who had not received any previous cancer therapy for their disease. Patients with the node-negative disease were included if they had primary tumors with a diameter of ≥2 cm. Other inclusion criteria were the availability of radiology (mammography, bilateral whole-breast and axillary sonography, and/or MR imaging), and pre-neoadjuvant and postsurgical pathological reports. Patients diagnosed with metastatic disease before the neoadjuvant treatment were excluded from the analysis. Patient and tumor characteristics included age at diagnosis, tumor size on imaging, clinical lymph node status, initial stage, immunohistochemistry (IHC) for hormone receptor expression of estrogen (ER) and/or progesterone receptors (PR) and HER2 expression evaluation via IHC and/or fluorescent *in situ* hybridization (FISH). In addition, information on the type of NST, type of surgery, and pathological determination of response to therapy have been collected. Pathological assessment was performed both on diagnostic pretreatment biopsy samples and surgical specimens of primary tumors. Axillary lymph node metastases were confirmed in all cases by ultrasound-guided fine-needle aspiration before the initiation of NST. Tumors were classified as hormone-receptor (HR) positive if ≥ 1% of tumor cells were stained for ER and/or PR. HER2+ status was defined by an immunohistochemistry score of 3 + or a HER2 amplification ratio of ≥2 by FISH. Physical examinations were performed every 3 weeks during systemic treatment. Patients underwent mammograms, ultrasound and/or MRI to assess tumor response before, during and after neoadjuvant therapy. At initial diagnosis, complete tumor staging was carried out, according to the tumor stage (chest X-ray and abdominal ultrasound, or CT scan or PET/CT scan). Diagnostic procedures were performed as required by local practice and national guideline [12]. The initial operability (breast-conserving surgery vs. mastectomy) as declared by the breast surgeon was recorded. If breast-conserving surgery was aimed at after NST (with the exception of multicentric or inflammatory tumors) the tumor was marked by placing a commercially available metal clip under ultrasound guidance. Multifocality or multicentricity of the tumor was not histologically verified in every case. The diagnosis of multifocality/multicentricity was based on radiologic imaging results. At diagnosis, before the initiation of NST, patients had to have an adequate cardiac function, with a left ventricular ejection fraction (LVEF) of at least 50% as measured by echocardiography. Echocardiography was performed prior to initiation of HER2 targeted therapy and then every 12 weeks in the adjuvant period according to the national guideline [12]. Laboratory parameters, blood counts and vital signs were assessed before each therapy and neutrophil-to-lymphocyte ratio (NLR) before the first chemotherapy. The pertuzumab loading dose was 840 mg, followed by 420 mg every 3 weeks. According to the label, the intravenous formulation of trastuzumab was used and administered every 3 weeks at a loading dose of 8 mg/kg, followed by doses of 6 mg/kg. After surgery, all patients received adjuvant conventional treatment with trastuzumab every 3 weeks to complete one year of therapy. Radiotherapy and standard hormone treatment for ER/PR+ (HR+) cases were prescribed according to the national guideline [12]. Patients with HR + tumors received tamoxifen or an aromatase inhibitor in combination with an LHRH-analogue, if premenopausal. As a primary endpoint of the study pCR was defined as the absence of invasive carcinoma at the microscopic examination in the breast and in the axillary lymph nodes at surgery (ypT0//is ypN0). Remaining *in-situ* lesions were allowed in the determination of pCR.

### Statistical Analysis

Associations between categorical outcome measures, such as stage and pCR were evaluated by chi2 or Fisher’s exact test. Eligibility for breast-conserving was scored as yes/no. ROC analysis of pCR (and separately for DFS) was performed to set the best cut-off values for continuous variables (age, stage, T, N, tumor size, Ki67, blood parameters and NLR). Multivariate logistic regression analysis was applied to examine the influence of parameters (e.g. tumor size, Ki67, tumor proliferation rate, HR status, and tumor grade) with *p* < 0.1 in univariate analysis on pCR. The survival analyses were performed for disease-free survival (DFS) and overall survival (OS) according to Kaplan-Meyer method. Multivariate Cox regression with factors predicting survival in univariate analyses (*p* < 0.1) was applied. The DFS was calculated from the beginning of neoadjuvant treatment until relapse, second primary, death caused by cancer or the last contact with the patient, whichever occurred first. The OS was considered from the start of neoadjuvant therapy until the death caused by cancer or last contact with the patient. All tests of hypotheses were two-sided. Results of statistical analyses were considered significant if *p* ≤ 0.05. Analyses were carried out by using the NCSS2019 Statistical Software (NCSS, LLC. Kaysville, Utah, USA, ncss. com/software/ncss).

## Results

We retrospectively identified 82 patients with stage II-III HER2+ EBC treated with neoadjuvant dual HER2 blockade of trastuzumab and pertuzumab in combination with chemotherapy who met the inclusion criteria for this analysis. The main patient and tumor characteristics are reported in Table 1. The vast majority of patients had an excellent performance status (ECOG 0: 91.9%). The clinical tumor status was cT1/2/3/4 in 9/55/12/6 patients and clinical nodal status was 0/1/2/3 in 13/52/13/4 patients. Fifty-five (67%) tumors were diagnosed at stage II and 27 (33%) tumors in stage III, and in three cases as inflammatory breast cancer. The majority of the tumors were of no special histological type. Retroareolar localization was observed in 14 (16.27%) cases. The most commonly used NST regimen consisted of a sequence of 3–4 cycles of anthracycline-based chemotherapy (EC or FEC) every 3 weeks followed by 3–4 cycles of a taxane (docetaxel) plus pertuzumab and trastuzumab. Only one patient was treated with 6 cycles of carboplatin plus docetaxel in combination with dual HER2-targeted therapy. In 4 cases (4.8%), the treatment consisted of 4 cycles of docetaxel in combination plus trastuzumab and pertuzumab given preoperatively, and these women received 3 cycles of an anthracycline combination (FEC) after surgery. Of the 82 patients, 42 received at least 3 cycles of trastuzumab plus pertuzumab. Seventy-nine patients (94%) received docetaxel as a taxane, and 39 out of 79 were treated with an initial dose of 100 mg/m^2^. In other cases, docetaxel was given at 75 mg/m^2^. In 2 cases, the taxane was paclitaxel administered either as a 3 weekly (175 mg/m^2^) or as a weekly schedule (80 mg/m^2^). Epirubicin was the most frequently used anthracycline, administered at a dose of 90 mg/m^2^.

**TABLE 1:** Parameters influencing the pCR. The bold values shows the value which keep its significance in multivariate analysis.

Parameters	N (%)	pCR (% of N)	*p* univariate	*p* multivariate
All	82 (100)	44 (54)		
Age (years), mean (range)	50.3 (27–77)			
<41	21 (26)	9 (43)	0.25	
≥41	61 (74)	35 (57)		
Side				
Left	31 (38)	17 (55)	0.938	
Right	50 (62)	27 (54)		
cSt				
IIa	19 (23)	11 (58)	0.673	
IIb-IIIc	63 (77)	33 (52)		
cT				
1	9 (11)	6 (67)	0.494	
2-4	73 (89)	38 (52)		
cN				
0–1	65 (79)	36 (55)	0.54	
2–3	17 (21)	8 (47)		
Size (mm), mean (range)	33.1 (10–60)			
<23	18 (22)	12 (67)	0.21	
≥23	64 (78)	32 (50)		
Multifocal/multicentric				
No	43 (52)	24 (56)	0.681	
Yes	39 (48)	20 (51)		
Location				
Central	14 (17)	7 (50)	0.763	
Non-central	68 (83)	37 (54)		
ER (%), mean (range)	25.7 (0–100)			
0	50 (61)	28 (56)	0.595	
≥1	32 (39)	16 (50)		
PR (%), mean (range)	9.96 (0–100)			
0	62 (76)	34 (55)	0. 706	
≥1	20 (24)	10 (50)		
HR				
−	47 (57)	26 (55)	0.727	
+	35 (43)	18 (51)		
Her2				
+++	67 (82)	38 (57)	0.241	
++/FISH+	15 (18)	6 (40)		
Ki67 (%), mean (range)	39.5 (0–80)			
<60	61 (74)	29 (48)	0.058	**0.015**
≥60	21 (26)	15 (71)		
Grade				
2	29 (35)	14 (48)	0.47	
3	53 (65)	30 (57)		
Histology				
Ductal	80 (98)	43 (54)	1	
Other	2 (2)	1 (50)		
Planned surgery				
Mastectomy	79 (96)	41 (52)	0.666	
Conservative	3 (4)	3 (100)		
Neoadjuvant cycles				
<7	43 (52)	21 (49)	0.357	
≥7	39 (48)	23 (59)		
Anthracyclin				
No	4 (5)	3 (75)	0.694	
Yes	78 (95)	41 (53)		
5FU				
No	33 (40)	15 (45)	0.221	
Yes	49 (60)	29 (59)		
Surgery				
Mastectomy	63 (77)	34 (54)	0.918	
Conservative	19 (33)	10 (53)		
Axillary dissection				
Yes	67 (82)	35 (52)	0.586	
No	15 (18)	9 (60)		
WBC (G/l), mean (range)	7.95 (3.5–18.3)			
<8.66	48 (67)	28 (58)	0.095	0.194
≥8.66	24 (33)	9 (38)		
NA	10	7		
Neutrophil (G/l), mean (range)	5.22 (1.44–13.1)			
<5.4	41 (58)	25 (61)	0.043	
≥5.4	30 (42)	11 (37)		
NA	11	8		
Lymphocyte (G/l), mean (range	2.05 (0.9–4.01)			
<1.62	13 (20)	4 (31)	0.121	
≥1.62	52 (80)	30 (58)		
NA	17	10		
Monocyte (G/l), mean (range)	0.51 (0.16–2.58)			
<0.63	54 (83)	31 (57)	0.099	
≥0.59	12 (17)	3 (25)		
NA	17	10		
NLR, mean (range)	2.68 (0.86–8.39)			
<1.976	15 (23)	11 (73)	0.081	0.044
≥1.976	50 (77)	23 (46)		
NA	17	10		

After the completion of neoadjuvant treatment (minimum of 4, and a maximum of 8 cycles), all patients underwent surgery three to five weeks after the last cycle of systemic therapy. The substantial majority of patients (95.2%) received all planned cycles preoperatively. A pCR (ypT0/is ypN0) was noted in 44 of 82 women (54%). The pCR rate was similar in HR- and HR + tumors. Concerning the stages of disease in patients who achieved pCR, the distribution was as follows: stage IIA 32% (6/19), stage IIB 58% (21/36), stage IIIA 67% (12/18), stage IIIB 100% (4/4) and for stage IIIC 20% (1/5). From the investigated factors Ki67 and NLR were significantly associated with pCR (Table 1) in multivariate analysis.

Out of 82 patients, for seventy-nine women (96.3%) mastectomy was planned and only 3 (3.7%) were candidates for breast conservation at diagnosis. After NST, a significant increase in conservative breast surgery rate (3.6% initially vs. 33% after NST, *p* = 0.0004) was observed. Axillary dissection was the standard approach for surgical staging of the axilla at that period, therefore only a minority of patients underwent a sentinel node biopsy (8 patients). A sentinel node biopsy (SLNB) before treatment was performed in one case with clinically negative axilla.

**FIGURE 1:**
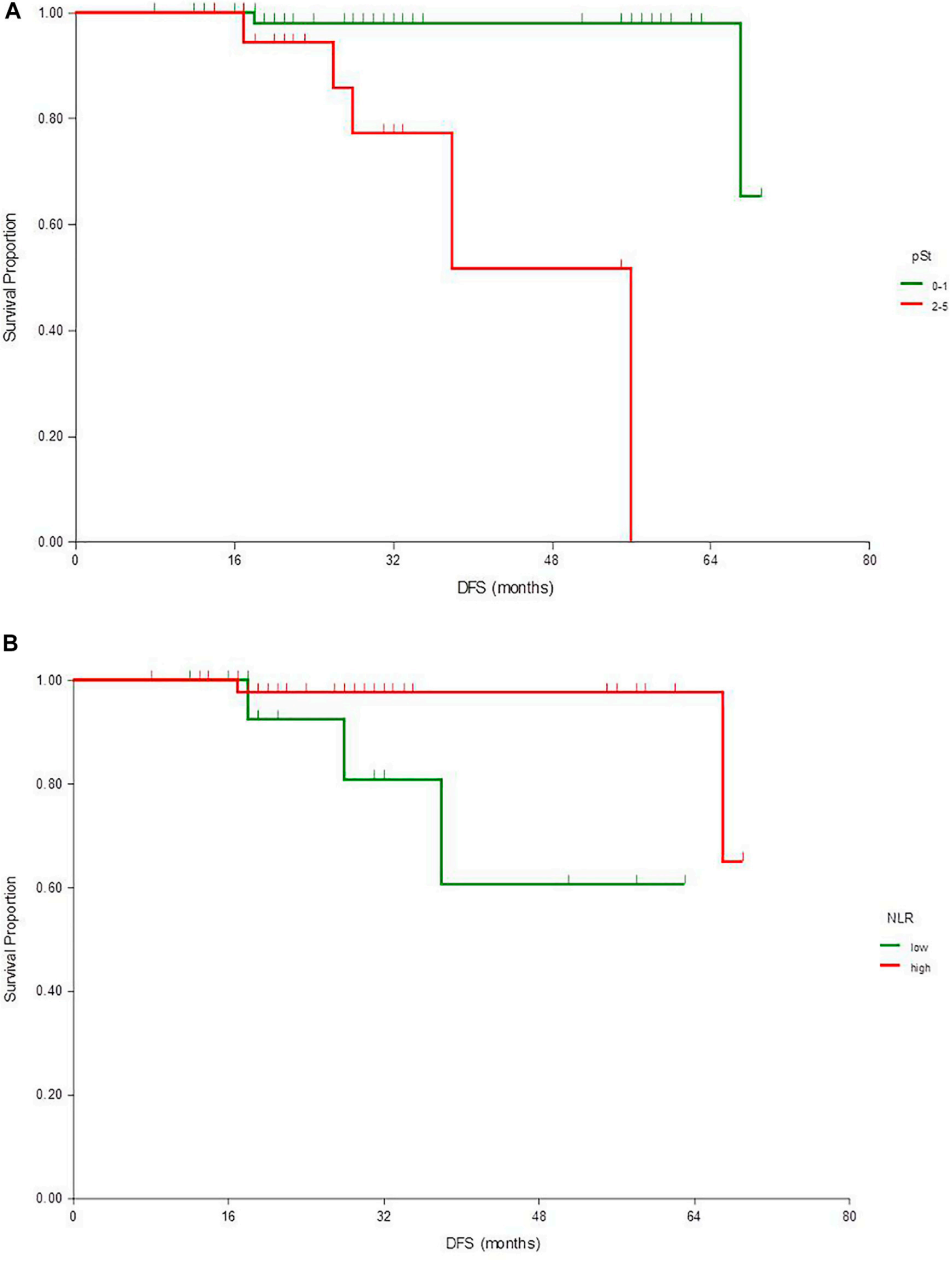
Kaplan-Meier estimates of disease-free survival according to pathological stage (**A**) and NLR (**B**)

After a median follow-up of 30 (95% CI 22–31) months, the median DFS and OS were not reached. The 2- and 5-years DFS rate was 97% (95% CI 93–100) and 82% (67–97), respectively. Disease relapse emerged in eight cases, two local and six distant metastases. Two patients deceased, one without known relapse, thus no further analysis was performed for OS. In univariate analysis pathologic status (ypT, ypN, ypStage and pCR), WBC and NLR had a significant association with DFS, although neither of these factors remained an independent prognostic factor in multivariate analysis (Table 2; Figure 1).

**TABLE 2:** Parameters influencing the DFS.

Parameters	N (%)	HR	*p* univariate	*p* multivariate
Age (years), mean (range)	50.3 (27–77)			
<39	11 (13)	0.26	0.146	
≥39	71 (87)			
Side				
Left	31 (38)	3.49	0.268	
Right	50 (62)			
cSt				
IIa-b	55 (67)	0.46	0.323	
IIIa-c	27 (33)			
cT				
1–2	64 (78)	0.54	0.477	
3–4	18 (22)			
cN				
0	13 (16)	0.28	0.189	
1–3	69 (84)			
Size (mm), mean (range)	33.1 (10–60)			
<45	65 (79)	0.21	0.068	0.208
≥45	17 (21)			
Multifocal/multicentric				
No	43 (52)	1.81	0.47	
Yes	39 (48)			
Location				
Central	14 (17)	1.54	0.732	
Non-central	68 (83)			
ER (%), mean (range)	25.7 (0–100)			
0	50 (61)	1.39	0.664	
≥1	32 (39)			
PR (%), mean (range)	9.96 (0–100)			
0	62 (76)	0.87	0.864	
≥1	20 (24)			
HR				
−	47 (57)	1.61	0.528	
+	35 (43)			
Her2				
+++	67 (82)	0.98	0.986	
++/FISH+	15 (18)			
Ki67 (%), mean (range)	39.5 (0–80)			
<40	44 (54)	0.52		
≥40	38 (46)			
Grade				
2	29 (35)	3.56	0.123	
3	53 (65)			
Histology				
Ductal	80 (98)	2.84	0.652	
Other	2 (2)			
Planned surgery				
Mastectomy	79 (96)	2.83	0.778	
Conservative	3 (4)			
Neoadjuvant cycles				
<7	43 (52)	0.35	0.196	
≥7	39 (48)			
Neoadjuvant + adjuvant cycles				
<7	39 (48)	0.48	0.443	
≥7	43 (52)			
Anthracyclin				
No	4 (5)	0.29	0.325	
Yes	78 (95)			
5FU				
no	33 (40)	1.59	0.564	
yes	49 (60)			
Surgery				
Mastectomy	63 (77)	0.63	0.621	
Conservative	19 (33)			
Axillary dissection				
Yes	67 (82)	3.62	0.17	
No	15 (18)			
ypT				
0–1	71 (87)	0.001	1.4 × 10^−4^	—
2–4	11 (13)			
ypN				
0	61(74)	0.03	5 × 10^−4^	—
1–2	21 (26)			
ypSt				
0-Ib	60 (73)	0.02	1.2 × 10^−4^	0.156
IIa-IIIb	22 (27)			
pCR				
Yes	38 (46)	0.12	0.015	—
No	44 (54)			
Adjuvant chemotherapy				
Yes	4 (5)	0.29	0.325	
No	78 (95)			
WBC (G/l), mean (range)	7.95 (3.5–18.3)			
<8.04	40 (56)	5.81	0.052	—
≥8.04	32 (44)			
NA	10			
Neutrophil (G/l), mean (range)	5.22 (1.44–13.1)			
<4.61	35 (49)	6.54	0.037	
≥4.61	36 (51)			
NA	11			
Lymphocyte (G/l), mean (range)	2.05 (0.9–4.01)			
<1.62	13 (20)	5.3	0.169	
≥1.62	52 (80)			
NA	17			
Monocyte (G/l), mean (range)	0.51 (0.16–2.58)			
<0.59	49 (75)	0.28	0.205	
≥0.59	16 (25)			
NA	17			
NLR, mean (range)	2.68 (0.86–8.39)			
<1.983	16 (25)	14.6	0.02	0.472
≥1.983	49 (75)			
NA	17			

## Discussion

As we look at newer therapies in oncology, the reproducibility of trial data in real-world populations is an increasing concern [13,14]. In line with Samiei et al. we observed that the primary breast tumor and axillary lymph node metastases responded similarly concerning the pattern and degree of response [15]. The result obtained is significant, neoadjuvant therapy based on dual blockade with pertuzumab and trastuzumab for HER2+ EBC in the routine clinical practice setting enabled the achievement of total pCR rates of 54%, which is comparable to the results from clinical trials. In Neosphere, Tryphaena and the more recent Kristine study, the pCR (ypT0/is and ypN0) rates were 39–52% [10,16,17]. High pCR rates were observed with dual targeting, regardless of patient demographics and tumor characteristics. The efficacy shown by the combination of pertuzumab and trastuzumab in our study was significant, even though the patients had worse prognostic characteristics; 22% percent of the tumors were ≥5 cm and a great number of cases showed nodal involvement (84%). In our analysis pCR rates were higher in ER-patients; however, the difference did not reach statistical significance. It is known that HER2+ tumors comprise a heterogeneous population where ER and/or PR expression occurs in ∼50% of the patients. Previous neoadjuvant studies reported lower pCR rates in HR + tumors as compared to HR-cases not reflected in survival analyses [10,16]. Furthermore, in biomarker analyses in the review article of Ischii and colleagues, only the HER2 expression level was predictive for efficacy, but not the HR-status [18]. However, in our analysis, HER2 expression level (++ or +++) has not significantly altered efficacy which could be explained with the small patient cohort. We could not find any correlation between pCR rates and patient or tumor characteristics beyond Ki67 and NLR; however, the number of patients was presumably too low to detect other statistically significant differences. Notably, the chemotherapy backbone of HER2-dual blockade in the neoadjuvant setting varied in daily practice. In this patient cohort anthracycline-, alkylator-, and taxane-based chemotherapy in combination with trastuzumab- and pertuzumab was the preferred treatment approach. Experience from our study showed that recommendations of international guidelines are followed in daily practice. Most of the patients involved in the study (95%) received all planned cycles before surgery. Almost 50% of the patients received a total of six treatment cycles, consisting of a sequence of 3 or 4 cycles of EC or FEC followed by 4 cycles of docetaxel given concomitantly with pertuzumab and trastuzumab. Non-anthracycline regimens (only one case in our cohort), such as docetaxel/carboplatin/trastuzumab/pertuzumab, have similar efficacy in the TRAIN-2 trial [19]. Its general application may be questioned due to cost coverage considerations.

NST has been widely used in EBC to downstage tumors, increase the rate of resection and eligibility for breast-conserving surgery. The higher rate of conversion from mastectomy to breast-conservative surgery with the addition of pertuzumab is not observed in previous trials, however, it is generally accepted that the rate of breast-conservation increased after neoadjuvant therapy [9,14,18]. A clear benefit of the application of neoadjuvant therapy in this study was that an increase in the breast-conserving surgery rate was achieved, resulting in a significantly higher proportion of patients who became eligible for breast-conserving surgery (3.6% vs. 33%, *p* = 0.0004). However, multifocal/multicentric disease and large tumor size were contributing factors leading to mastectomy and discordance between high pCR rates and low breast-conserving surgery rates. Interestingly in our study population, a high percentage of multifocal/multicentric tumors (41/86) was observed, although the multifocality or multicentricity was not verified with biopsy (multifocal and multicentric tumors were defined as two or more synchronous ipsilateral neoplasms, in the same or different quadrants of the breast).

Furthermore, some patients eligible for breast-conserving surgery opted for mastectomy. Our result is in line with previous reports, although Gonzales et al. reported a higher percentage of breast-conservation [20]. It may also be influenced by other factors than resectability [21].

The NLR is widely investigated as a prognostic factor in breast cancer. Also in HER2+ early disease, it has an independent prognostic role in retrospective investigations [22]. Bae et al. reported in a neoadjuvant setting that NLR is prognostic with trastuzumab plus chemotherapy but not when pertuzumab is added [23]. In our analysis lower NLR was an independent predictor of pCR, but there was no significant association with DFS in multivariate analysis. Interestingly, we detected even worse survival with lower NLR, for which phenomenon we do not have an explanation, but it can underline the fact that in this patient population treated with dual anti-HER2 blockade NLR is not a useful marker for therapy planning.

In recent years there has been a shift toward more preoperative systemic treatment in the management of early stage HER2+ EBC. NST for HER2+ EBC is becoming even more important with an increasing number of post-neoadjuvant treatment options. Depending on the results achieved with NST post-neoadjuvant therapy, further tailored treatment procedures based on the extent of residual disease can be recommended.

Evidence from clinical trials supports the neoadjuvant use of pertuzumab and trastuzumab with chemotherapy to improve pCR in HER2+ EBC, but information on the efficacy of this combination in the clinical setting is still limited. Overall, treatment with pertuzumab and trastuzumab in combination with chemotherapy was generally well-tolerated, and no treatment discontinuation due to side effects was observed in our investigation. The reproducibility of trial data in daily practice is of increasing concern because they also reflect the actual clinical environments in which treatments are used, as well as their results. Nevertheless, the major strength of this analysis is in its representation of a real-world population and our study has revealed important aspects of clinical practice.

Our study had limitations, the first of which was the retrospective nature of our study design. We did not collect data on neoadjuvant therapy without pertuzumab, therefore comparison has not been carried out on treatment with and without pertuzumab. In HER2+ breast cancer with neoadjuvant treatment, there are other putative prognostic factors not included in this analysis (tumor-infiltrating lymphocytes, PI3KCA mutation), which may influence the result of prognostic factor evaluation. Toxicity data were only partially available for our patient population due to the retrospective study design applied to the real-world setting and collecting safety data was not the scope of this study.

In conclusion, the daily experience in practice showed a meaningful clinical activity of neoadjuvant pertuzumab and trastuzumab with chemotherapy in improving the pCR rates in HER2+ EBC and demonstrated the reproducibility of clinical trial data in the real‐life setting. Consistent with previous studies, we did not find a biomarker suitable for the selection of patients who should not receive pertuzumab in addition to neoadjuvant chemotherapy and trastuzumab. The novelty of this study was that we could demonstrate a high pCR rate within the daily experience with dual HER2-targeted therapy which led to a significantly increased rate of breast-conserving surgery.

## Data Availability

The raw data supporting the conclusions of this article will be made available by the authors, without undue reservation.
